# Revisiting the genetic diversity and population structure of the endangered Green Sea Turtle (*Chelonia mydas*) breeding populations in the Xisha (Paracel) Islands, South China Sea

**DOI:** 10.7717/peerj.15115

**Published:** 2023-03-22

**Authors:** Meimei Li, Ting Zhang, Yunteng Liu, Yupei Li, Jonathan J. Fong, Yangfei Yu, Jichao Wang, Hai-tao Shi, Liu Lin

**Affiliations:** 1 Ministry of Education Key Laboratory for Ecology of Tropical Islands, Key Laboratory of Tropical Animal and Plant Ecology of Hainan Province, College of Life Sciences, Hainan Normal University, Haikou, China; 2 Hainan Sansha Provincial Observation and Research Station of Sea Turtle Ecology, Sansha, China; 3 Marine Protected Area Administration of Sansha City, Sansha, China; 4 Science Unit, Lingnan University, Hong Kong SAR, China

**Keywords:** Genetic diversity, Green sea turtle, Mitochondrial DNA, Xisha (Paracel) Islands

## Abstract

The Green Sea Turtle (*Chelonia mydas*) is an umbrella species in the South China Sea, a Chinese national first-level protected wild animal, and the only sea turtle that nests in waters around China. The largest *C. mydas* nesting ground is distributed in the Xisha (Paracel) Islands, which plays a vital role in the survival of sea turtle populations in the region. This study reveals the genetic diversity and population structure of the breeding population of *C. mydas* in the Xisha (Paracel) Islands using three mitochondrial markers. A total of 15 D-loop, five Cytochrome b (Cyt b), and seven Cytochrome C Oxidase subunit I (COI) haplotypes were identified in the breeding population of *C. mydas* in the Xisha (Paracel) Islands. D-loop haplotypes are distributed in clades III, IV, and VIII of the *C. mydas* mitochondrial control region. It is the first time that one haplotype from Clade IV was found in this *C. mydas* population, and five new D-loop haplotypes were also identified. The haplotype and nucleotide diversity were calculated for each marker: D-loop (0.415 haplotype diversity, 0.00204 nucleotide diversity), Cyt b (0.140, 0.00038) and COI (0.308, 0.00083). The average genetic distance (*p*) of each molecular marker was less than 0.01. Neutral detection and nucleotide mismatch analysis suggested that the breeding population of *C. mydas* in the Xisha (Paracel) Islands did not experience a population expansion event in recent history. It is recommended that a sea turtle protection area be established in the Xisha (Paracel) Islands area to strengthen protection and effectively protect the uniqueness and sustainability of the breeding population of *C. mydas* in the South China Sea.

## Introduction

Sea turtles are large marine migratory reptiles, which are widely distributed in the Pacific, Indian, and Atlantic Ocean warm water (Hirth, 1997). As marine flagship species, sea turtles are vital for maintaining the health of marine ecosystems and are also important indicator species for marine environmental monitoring (Bouchard & Bjorndal, 2000; Hamann et al., 2010). Five sea turtle species inhabit seas around China, and over 90% of the population is distributed in the South China Sea (Wang, 1993; Jiang et al., 2000; Mou et al., 2013). However, sea turtle populations have decreased sharply in China owing to coastal zone development (Chan et al., 2007), illegal commercial trade (Lam et al., 2011), marine pollution, and climate change (Wabnitz et al., 2018). As a result, all five sea turtle species have been upgraded from level II to level I on the “List of Wildlife under Special State Protection” of China (Lin et al., 2021).

The Green Sea Turtle (*Chelonia mydas*) is the only sea turtle species that lays eggs in seas around China (Wang et al., 2019). Its nesting grounds were once widely distributed in China’s southern coastal areas in Hainan, Guangxi, Guangdong, and Fujian Provinces. However, most of these nesting grounds have disappeared in the last century, including in the Huidong Sea Turtle National Nature Reserve, which has had no recorded nests since 2018 (Lin et al., 2021). The Xisha (Paracel) Islands are currently the largest nesting grounds for *C. mydas* in seas around China, with more than 100 nests recorded annually (Wang et al., 2019; Jia et al., 2019). Recent studies have found that *C. mydas* from the Xisha (Paracel) Islands rookery represent a new geographic population with unique haplotypes and a new conservation management unit with a high conservation value (Gaillard et al., 2021; Song et al., 2022).

Genetic diversity is an important feature of a species, reflecting its potential to adapt to environmental change (Ekanayake et al., 2017). Sea turtles are long-lived organisms, and their life histories are marked by ontogenic habitat shifts and large-scale migrations (Bowen & Karl, 2007). Therefore, accurately and comprehensively understanding sea turtle genetic diversity will help develop effective conservation strategies. Due to its simple structure and relatively rapid evolution rate, mitochondrial DNA (mtDNA) molecular markers are the commonly used genetic markers for assessing sea turtle population structure, genetic diversity, and phylogeography (Naro-Maciel et al., 2008; Guo, Wang & Liu, 2009; Leroux et al., 2012; Vargas et al., 2016; Yang, 2015). Yang (2015) and Wei (2016) both used mitochondrial genes to do a preliminary study of the genetic diversity of *C. mydas* in the South China Sea, but their samples were mainly juvenile sea turtles from eggs that were incubated artificially or from *C. mydas* confiscated by the coast guard. Unclear sample collection site may not reflect the level of genetic diversity in the wild breeding population of *C. mydas* in South China Sea. Gaillard et al. (2021) and Song et al. (2022) also studied breeding populations of *C. mydas* in the Xisha (Paracel) Islands, but their sample sizes were small (*n* = 16 or *n* = 13), limiting the application of the data to resource management and conservation of *C. mydas* breeding populations in this area.

From 2017 to 2021, a total of 72 individual samples were collected from the *C. mydas* nesting grounds in the Xisha (Paracel) Islands. This study aimed to (1) comprehensively evaluate the genetic diversity of the breeding population of *C. mydas* in the Xisha (Paracel) Islands using the three mtDNA markers and understand the evolutionary and adaptive potential of *C. mydas* in this region and (2) analyze the genetic structure and historical dynamics of the population. This work will provide a scientific basis for management activities of the *C. mydas* breeding population inhabiting the Xisha (Paracel) Islands.

## Materials and Methods

### Sampling

**Figure 1:**
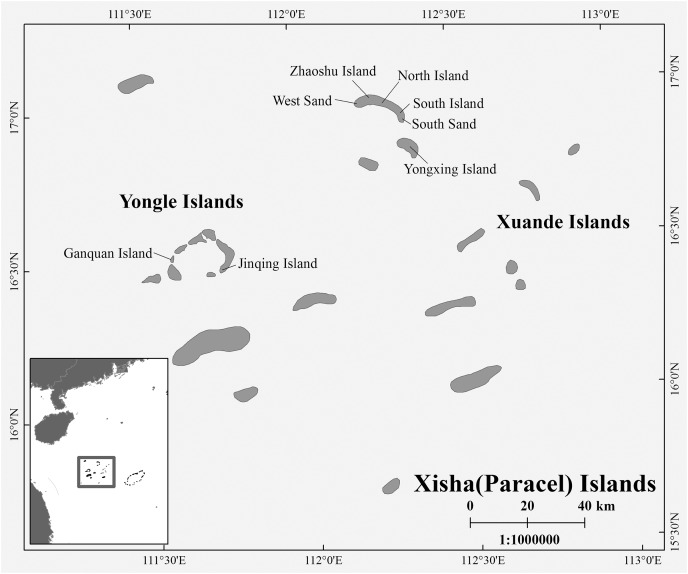
Map of study area and sampling points. Sampling points West Sand; North Island; South Island; South Sand; Ganquan Island; Jinqing Island.

During the breeding season of *C. mydas* in the Xisha (Paracel) Islands of the South China Sea, biopsy punch sampling methods were used to collect hind limbs skin samples from female turtles that came ashore to lay eggs. Biopsy sites were disinfected with iodophor (LIRCON, Shandong, China) before and after sampling. After the female turtle lays eggs, we used the scanner (RBC-S03; Raybaca IOT Technology, Anhui, China) to identify whether the female turtle has Passive Integrated Transponder (PIT) markers (RBC-Z00; Raybaca IOT Technology, Anhui, China), and make chip markers and samples for the newly discovered female turtle, so as to ensure that the sampling of female turtles was not repeated. In addition to nesting females, we also took samples from already-hatched nests, either from the forelimb skin of hatchlings or from embryos that died in the nest. The duration of each field sampling work on one site did not exceed 20 days, so the possibility that hatchling or embryos came from the collected female turtles was small. Between 2017 and 2021, a total of 72 samples (Yongle Islands: 12 samples; Xuande Islands: 60 samples) were collected, and all samples were stored in 95% alcohol at −20 °C (Table S1 and Fig. 1). The sample collection work was approved by the Chinese government, and this work was conducted in strict accordance with the guidelines of the Animal Research Ethics Committee of Hainan Provincial Education Centre for Ecology and Environment, Hainan Normal University (HNECEE-2012-005).

### Laboratory work

DNA was extracted from skin samples using a blood/cell/tissue genomic DNA extraction kit (DP304; Tiangen Biotech Co., Ltd., Beijing, China). DNA concentrations were all above 4 ng/μl.

Three sets of primers were used to amplify the target sequences of the control region (D-loop), Cytochrome b (Cyt b), and Cytochrome C Oxidase subunit I (COI) (Table 1).

**Table 1:** Mitochondrial DNA primers for *Chelonia mydas*.

Marker	Primer	Sequence	Product length (bp)	Reference
D-loop	LCM15382	GCTTAACCCTAAAGCATTGG	770	Abreu-Grobois et al. (2006)
	H950g	AGTCTCGGATTTAGGGGTTTG		
Cytb	Cytb-f	ATTCTTGCCTGGACTTTA	1,140	Yang (2015)
	Cytb-r	TTCAATCTTTGGTTTACA		
COI	COI-f	TCAACCAACCACAAAGACATTGGCAC	650	Yang (2015)
	COI-r	TAGACTTCTGGGTGGCCAAAGAATCA		

PCR was performed in 50 µl reactions, with 25 µl of 2×Taq Mix (RN03001S, MonAmp™), 2 µl of template DNA, 2 µl of each 10 uM primer, and ultrapure water to 50 µl. The PCR conditions of D-loop as follows: pre-denaturation at 94 °C for 3 min; followed by 30 cycles of denaturation at 94 °C for 30 s, annealing at 51 °C for 30 s, extension at 72 °C for 60 s; and final extension at 72 °C for 3 min. The PCR conditions of Cyt b as follows: pre-denaturation at 94 °C for 80 s; followed by 30 cycles of denaturation at 94 °C for 42 s, annealing at 47 °C for 30 s, extension at 72 °C for 80 s; and final extension at 72 °C for 5 min. The PCR conditions of COI as follows: pre-denaturation at 94 °C for 80 s; followed by 30 cycles of denaturation at 94 °C for 42 s, annealing at 55 °C for 30 s, extension at 72 °C for 80 s, and final extension at 72 °C for 5 min. PCR products were checked with a 1% agarose gel and successful reactions purified and Sanger sequenced using PCR primers at Guangzhou Ige Biotechnology Co., Ltd.

### Data analysis

#### Sequence composition analysis

D-loop, Cyt b, and COI Sequences were spliced using Sequencher 5.4.5 software (Sequencher® version 5.4.5 DNA sequence analysis software, http://www.genecodes.com; Gene Codes Corporation, Ann Arbor, MI, USA). ClustalW program of MEGA-X software’s (Kumar et al., 2018) was used to align the sequences and calculate the number of parsimony informative sites and variable sites. Default values of parameters were used in the analysis.

#### Haplotype and genetic diversity analysis

Shorter control region segments were commonly used to align and analyze population structures in *C. mydas*, in particular a 384 bp region found in the Indo-Pacific region. Accordingly, the ~753 bp sequences obtained were subsequently trimmed to 384 bp for further comparison with the available data using the standardized CmP nomenclature.

BLAST was used to compare sequences to GenBank to identify existing haplotypes or confirm new haplotypes. DnaSP v5 software (Librado & Rozas, 2009) was used to detect the number of haplotypes, nucleotide, haplotype diversity, and average number of nucleotide differences.

#### Population genetic differentiation analysis

MEGA-X was used to align the sequences and calculate the genetic distance of each haplotype. Genetic differentiation index Fst and gene flow (Nm) between two geographic populations (Xuande, Yongle) were obtained using DnaSP5.0 software based on sequences data. The software Network10.2 (Polzin & Vahdati, 2003) was used to draw the haplotype network diagram, which reflected the evolutionary relationship among haplotypes.

IQ-TREE (Trifinopoulos et al., 2016) was used to determine the best-fit model of nucleotide substitutions. The software MrBayes v3.1.2 (Huelsenbeck & Ronquist, 2001) was used to perform Bayesian phylogenetic reconstructions (ngen = 20,000,000, printfreq = 100, samplefreq = 100, nchains = 4, and burnin = 50,000). The MEGA-X software was used to construct a phylogenetic tree based on the Hasegawa-Kishino-Yano model with the Maximum Likelihood method (ML), including 11 D-loop haplotypes from five evolutionary clades of *C. mydas* distributed in the Indo-Pacific control region (Jensen et al., 2019), and the *Eretmochelys imbricata* (GenBank: AJ421794) as an outgroup (File S1). The confidence of each branch of the ML tree was tested by 1,000 bootstrapping repetitions, retaining the default values for other parameters.

#### Population dynamic history analysis

For each of the two populations (Xuande, Yongle), Fu’s Fs and Tajima’s D neutrality tests and mismatch distribution analysis were carried out using DnaSP v5 software.

## Results

### Sequence composition

The number of new sequences obtained from the Xisha (Paracel) Islands breeding population for each marker was as follows: 72 D-loop (753 bp length), 69 Cyt b (1,052 bp), and 71 COI (511 bp). The parsimony informative sites and variable sites were calculated for each marker: D-loop (seven parsimony informative sites, 38 variable sites), Cyt b (2, 12), and COI (4, 6).

### Haplotype and genetic diversity

A total of 12 D-loop, five Cyt b, and seven COI haplotypes were identified from the 72 samples collected from the nesting grounds of the Xisha (Paracel) Islands (Table 2). The haplotype diversity (*h*) of each marker ranged from 0.140 to 0.415, while the nucleotide diversity (*n*) ranged from 0.00038 to 0.00204 (Table 3). Based on the cut-off point of haplotype diversity (0.5) and nucleotide diversity (0.005) set by Grant & Bowen (1998), the breeding population of *C. mydas* in the Xisha (Paracel) Islands showed low haplotype diversity and nucleotide diversity. The haplotype diversity of the D-loop was 0.415 ± 0.07, slightly lower than the mean value found for the Indo-Pacific *C. mydas* management units (mean 0.469 ± 0.07). The nucleotide diversity was 0.00204 ± 0.00, lower than the Indo-Pacific *C. mydas* mean of 0.0084 ± 0.00 (Table 4) (Dethmers et al., 2006; Cheng et al., 2008; Nishizawa et al., 2011; Jensen et al., 2016).

**Table 2:** D-loop haplotypes and frequencies of *Chelonia mydas* from the Xisha (Paracel) Islands.

Haplotype	Samples	Frequency (%)	Samples	Frequency (%)
	Xuande Islands		Yongle Islands	
D-loop	60		12	
CmP18.1	1	1.7	1	8.3
CmP19.1	46	76.7	9	75
CmP47.1	0	0	1	8.3
CmP49.1	1	1.7	1	8.3
CmP57.1	1	1.7	0	0
CmP75.1	1	1.7	0	0
CmP154.1	4	6.7	0	0
CmP250.1*	1	1.7	0	0
CmP251.1*	1	1.7	0	0
CmP252.1*	2	3.3	0	0
CmP253.1*	1	1.7	0	0
CmP254.1*	1	1.7	0	0
Cytb	57		12	
CMB1*	53	93	11	91.7
CMB2*	2	3.5	0	0
CMB3*	0	0	1	8.3
CMB4*	1	1.8	0	0
CMB5*	1	1.8	0	0
COI	59		12	
CMC1	50	84.7	9	75
CMC2*	2	3.4	0	0
CMC3*	1	1.7	1	8.3
CMC4*	1	1.7	0	0
CMC5	3	5.1	1	8.3
CMC6*	2	3.4	0	0
CMC7	0	0	1	8.3

**Note:**

* Represents the haplotype newly identified in this study.

**Table 3:** The genetic diversity parameters and the haplotype average genetic distance of *Chelonia mydas* individuals of this study, separated by Island group.

Marker	Group	# Samples	# Haplotypes	Haplotype diversity	Nucleotide diversity	Average nucleotide difference number (*k*)	Average genetic distance (*p*)	Variable sites	Parsimony informative sites
D-loop	Xuande	60	11	0.411 ± 0.080	0.00099 ± 0.000	0.740			
	Yongle	12	4	0.455 ± 0.170	0.00733 ± 0.003	5.470			
	Total	72	12	0.415 ± 0.074	0.00204 ± 0.002	1.522	0.010	38	7
Cyt b	Xuande	57	4	0.136 ± 0.061	0.00030 ± 0.000	0.313			
	Yongle	12	2	0.167 ± 0.134	0.00079 ± 0.000	0.833			
	Total	69	5	0.140 ± 0.057	0.00038 ± 0.000	0.404	0.0046	12	2
COI	Xuande	59	6	0.281 ± 0.076	0.00070 ± 0.000	0.359			
	Yongle	12	4	0.455 ± 0.170	0.00146 ± 0.001	0.742			
	Total	71	7	0.308 ± 0.071	0.00083 ± 0.000	0.426	0.0040	6	4

**Table 4:** Comparison of genetic diversity of D-loop marker between the Xisha (Paracel) Islands and other locations in the Indo-Pacific region.

Country	Management unit	# Haplotypes	Haplotype diversity (*h*) ± SD	Nucleotide diversity (*n*) ± SD	# Samples
China	Xisha Islands	12	0.415 ± 0.07	0.002 ± 0.00	72
China	Eastern Taiwan^C^	1	0.000 ± 0.00	0.000 ± 0.00	14
China	Western Taiwan^C^	3	0.483 ± 0.06	0.028 ± 0.01	40
Indonesia	Western Java^A^	2	0.485 ± 0.06	0.001 ± 0.00	22
Indonesia	Aru^A^	2	0.071 ± 0.07	0.004 ± 0.00	28
Indonesia	Sangalaki^D^	5	0.780 ± 0.16	0.008 ± 0.03	29
Malaysia	Sipadan^A^	7	0.630 ± 0.07	0.005 ± 0.00	98
Malaysia	Peninsula Malaysia^A^	7	0.567 ± 0.10	0.008 ± 0.00	29
Malaysia	Sarawak^A^	3	0.450 ± 0.11	0.009 ± 0.00	22
Malaysia and Philippines	Turtle Islands^A^	3	0.369 ± 0.06	0.001 ± 0.00	66
Australia	Cocos Keeling Island^A^	2	0.199 ± 0.11	0.011 ± 0.01	19
Australia	Ashmore Reef^A^	7	0.670 ± 0.04	0.030 ± 0.02	44
Australia	Scott Reef and Browse Island^A^	5	0.498 ± 0.06	0.007 ± 0.00	64
Australia	North West Shelf^A^	8	0.433 ± 0.07	0.004 ± 0.00	76
Australia	Cobourg Peninsula^A^	5	0.574 ± 0.08	0.003 ± 0.00	37
Australia	Gulf of Carpentaria^A^	6	0.637 ± 0.02	0.004 ± 0.00	127
Japan	Ogasawara^B^	13	0.706 ± 0.04	0.018 ± 0.01	103
Mean		5.35	0.469 ± 0.07	0.0084 ± 0.00	52.35

Notes:

A Jensen et al. (2016).

B Nishizawa et al. (2011).

C Cheng et al. (2008).

D Dethmers et al. (2006).

Among the 12 D-loop haplotypes, seven were found in previous studies (CmP18.1, CmP19.1, CmP47.1, CmP49.1, CmP57.1, CmP75.1 and CmP154.1) (Dutton et al., 2014; Gaillard et al., 2021; Song et al., 2022), while five were newly discovered (CmP250.1, CmP251.1, CmP252.1, CmP253.1 and CmP254.1, with GenBank accession numbers OK284742, OK324138, OP320709, OP320710, and OP320711, respectively), and they were all found in the Xuande Islands. The most common haplotype in the samples was CmP19.1, accounting for 76.4% of the total samples from the nesting grounds of the Xisha (Paracel) Islands, followed by CmP154.1, accounting for 5.6% and CmP18.1, CmP49.1, and CmP252.1, each accounting for 2.8%. All five Cyt b haplotypes were newly discovered (CMB1–CMB5) and haplotype CMB1 was the most common, with a frequency of 92.75%. For the seven COI haplotypes, four (CMC2, CMC3, CMC4, and CMC6) were newly discovered in this study. Among them, haplotype CMC1 was the most common, with a frequency of 83.1%, and the other haplotypes were relatively rare.

### Population genetic differentiation

Of the breeding population of *C. mydas* in the Xisha (Paracel) Islands, the average genetic distances of the D-loop, Cyt b, and COI haplotypes were 0.010, 0.0046, and 0.0040, respectively (Table 3). The genetic distance between D-loop haplotype CmP47.1 and the other 11 haplotypes was the largest (0.04), with most of the genetic distances between 0.001 and 0.008. The genetic distances between the haplotypes of the Cyt b and COI genes ranged from 0.001 to 0.009 and 0.002 to 0.006, respectively.

The genetic differentiation coefficient Fst between the two geographic groups of *C. mydas* in Xuande and Yongle Islands was −0.00690 to 0.01303, and the gene flow coefficient (Nm) was −36.46 to 114.37 (Table 5), indicating that there was no genetic differentiation between the two geographic populations and gene exchange was frequent.

**Table 5:** Fst values and gene flow between two geographical groups of *Chelonia mydas* in Xuande Islands and Yongle Islands.

Marker	Fst	Gene flow (Nm)
D-loop	−0.00690	−36.46
Cyt b	0.00218	114.37
COI	0.01303	18.93

Combining the seven 384 bp D-loop haplotypes (CmP18, CmP19, CmP20, CmP49, CmP54, CmP75, and CmP244) found in Gaillard et al. (2021) and Song et al. (2022), there were, in total, 15 D-loop haplotypes in the breeding population of *C. mydas* in the Xisha (Paracel) Islands (Table 6).

**Table 6:** The clade, haplotypes (384 bp control region) and samples number of *Chelonia mydas* in Xisha (Paracel) Islands, South China Sea (Clade reference Jensen et al., 2019).

Clade	Haplotype	Samples	Reference
III	CmP20	1	Song et al. (2022)
	CmP54	1	Gaillard et al. (2021)
	CmP244	1	Song et al. (2022)
IV	CmP47	1	This study
VIII	CmP18	4	Song et al. (2022) Gaillard et al. (2021) This study
	CmP19	73	Song et al. (2022) Gaillard et al. (2021) This study
	CmP49	6	Gaillard et al. (2021) This study
	CmP57	1	This study
	CmP75^#^	1	This study
	CmP154	4	This study
	CmP243^#^	2	Song et al. (2022)
	CmP250	1	This study
	CmP251	1	This study
	CmP252	2	This study
	CmP253	1	This study
	CmP254	1	This study
Total		101	

**Note:**

# The same haplotype. CmP243 is actually the same haplotype as CmP75.

**Figure 2:**
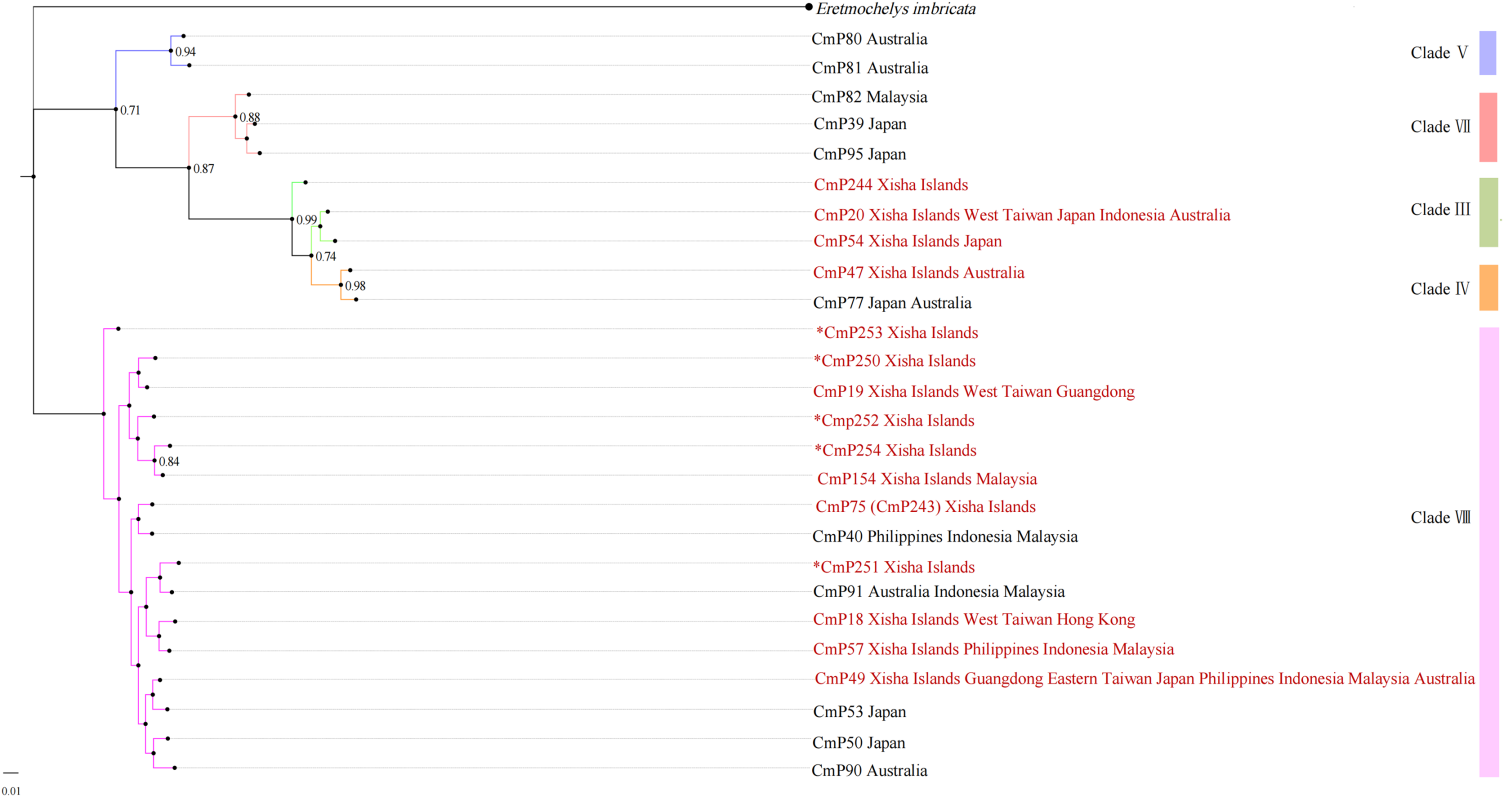
Bayesian tree of 26 D-loop haplotypes (384 bp) from rookeries of *Chelonia mydas* in the Indo-Pacific and Japan regions, including 15 haplotypes from the Xisha (Paracel) Islands (red text). CmP243 is actually the same haplotype as CmP75. The posterior probability greater than 0.70 is labeled next to the corresponding branches. The clades used here follow Jensen et al. (2019). An asterisk (*) indicates the five new haplotypes identified in this study (Ng et al., 2017; Jensen et al., 2019; Gaillard et al., 2021; Song et al., 2022; this study).

With reference to the clades studied by Jensen et al. (2019), the Xisha (Paracel) Islands D-loop haplotypes belonged to three clades, with three haplotypes (CmP20, CmP54, and CmP244) belonging to clade III, one haplotype (CmP47) belonging to clade IV, and the other 11 haplotypes belonging to clade VIII (Table 6 and Fig. 2). There was no obvious difference between the topology of Bayesian tree and Maximum Likelihood tree (Fig. S1).

**Figure 3:**
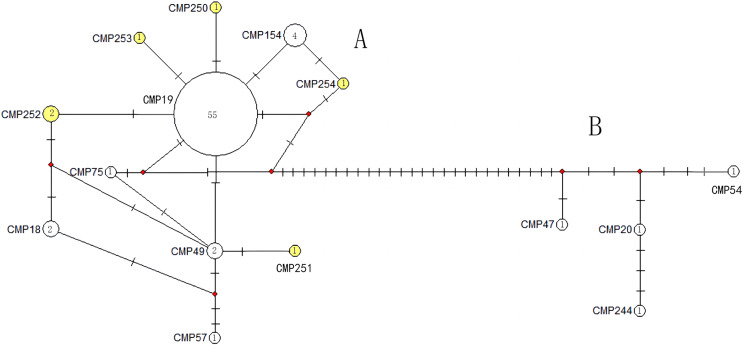
The haplotype network diagram showing the relationships between the 15 D-loop haplotypes among the *Chelonia mydas* in Xisha (Paracel) Islands. Number of mutations between haplotypes are illustrated by dashes in connecting lines; the red dots indicate the missing haplotype in the middle; the yellow circles represent the new haplotypes identified in this study; numbers in the circles indicated sample size of each haplotype. The haplotypes in cluster A all distribute in clade VIII, and haplotypes in cluster B distribute in clades III and IV (Jensen et al., 2019).

**Figure 4:**
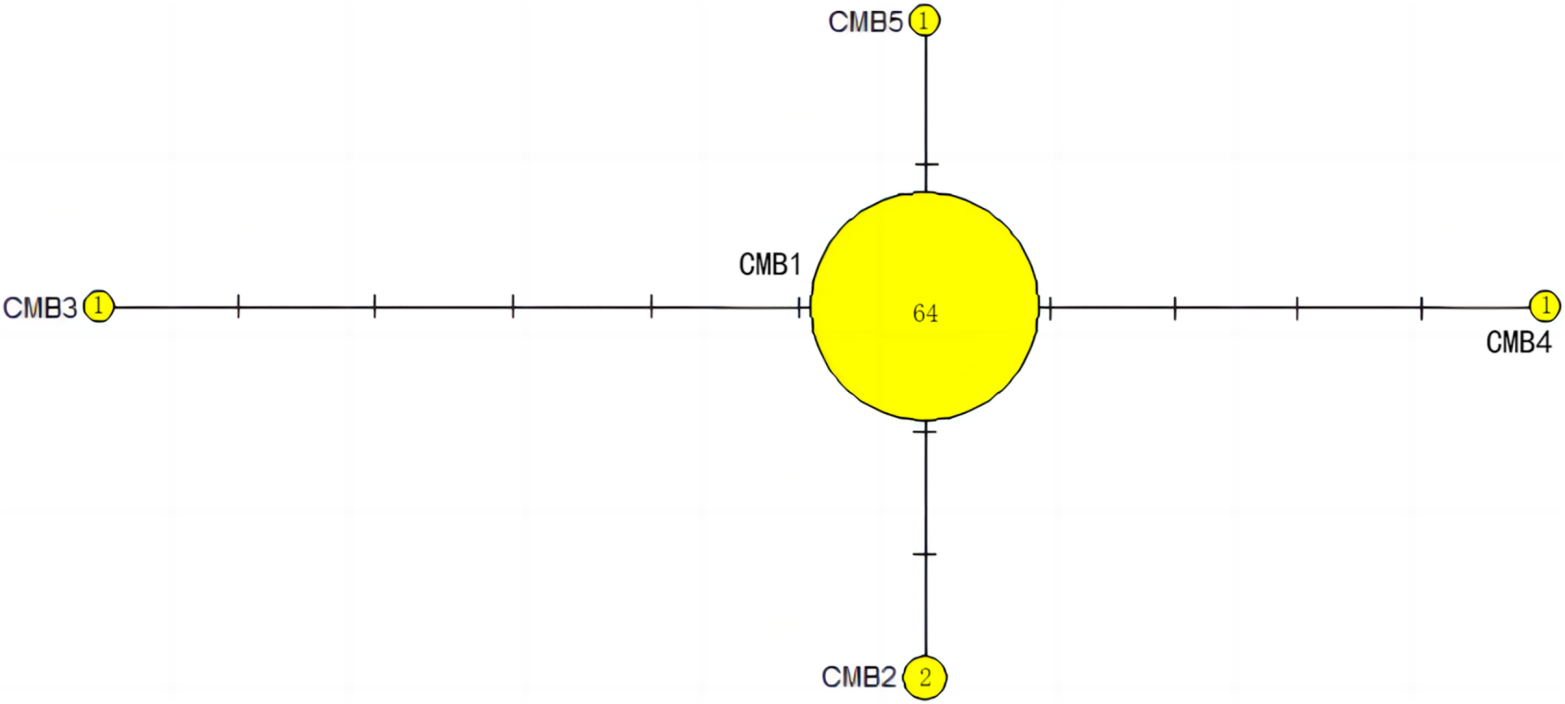
The haplotype network diagram showing the relationships between the five Cyt b haplotypes among the *Chelonia mydas* in Xisha (Paracel) Islands. The number of mutations between haplotypes are illustrated by dashes in connecting lines. The yellow circles represent new haplotypes identified in this study. The numbers in the circles indicated sample size of each haplotype.

**Figure 5:**
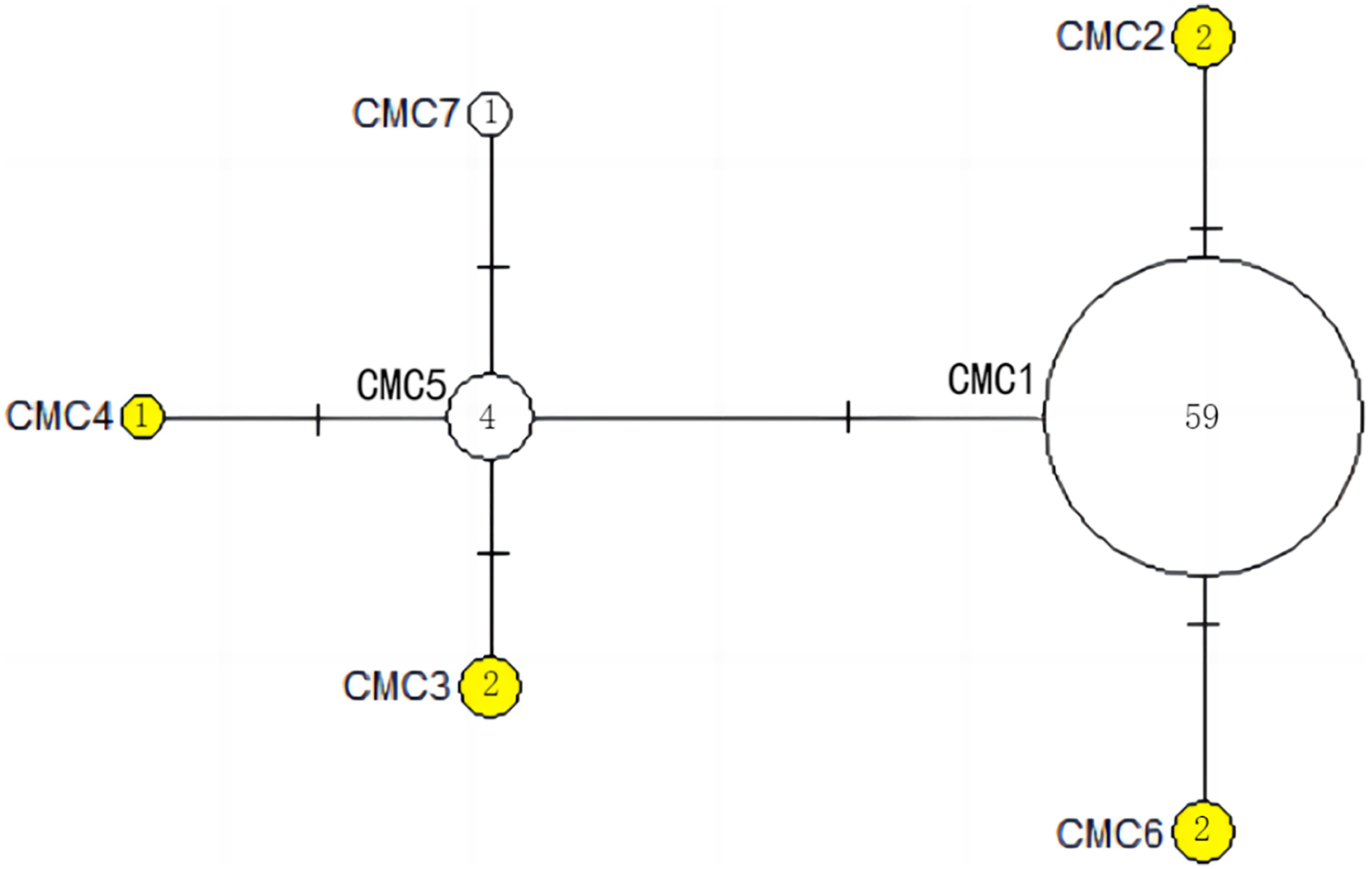
The haplotype network diagram showing the relationships between the seven COI haplotypes among the *Chelonia mydas* in Xisha (Paracel) Islands. The number of mutations between haplotypes are illustrated by dashes in connecting lines. The yellow circles represent new haplotypes identified in this study. The numbers in the circles indicated sample size of each haplotype.

The haplotype network diagrams (Figs. 3–5) reflected the evolutionary relationship among haplotypes of various genes of *C. mydas* in the Xisha (Paracel) Islands. As showned in Fig. 3, the D-loop haplotype network was mainly divided into two clusters (A and B), a result largely consistent with the Bayesian tree topology. The haplotypes in cluster A all distributed in clade VIII and haplotypes in cluster B distributed in clades III and IV (Jensen et al., 2019). The haplotype network diagram of Cyt b haplotypes showed radial divergence, with the most frequent haplotype, CMB1, as the central haplotype. The haplotype network of COI haplotypes was also radial, with the most frequent haplotype, CMC1.

### Population dynamic history

**Figure 6:**
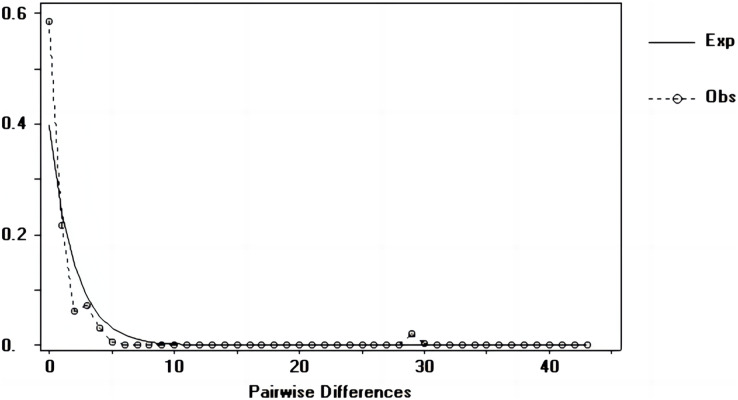
Nucleotide mismatch distributions of the D-Loop markers used in this study.

**Figure 7:**
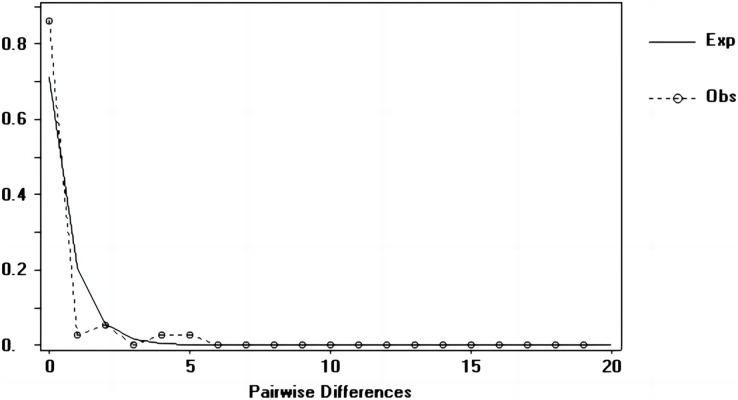
Nucleotide mismatch distributions of the Cyt b markers used in this study.

**Figure 8:**
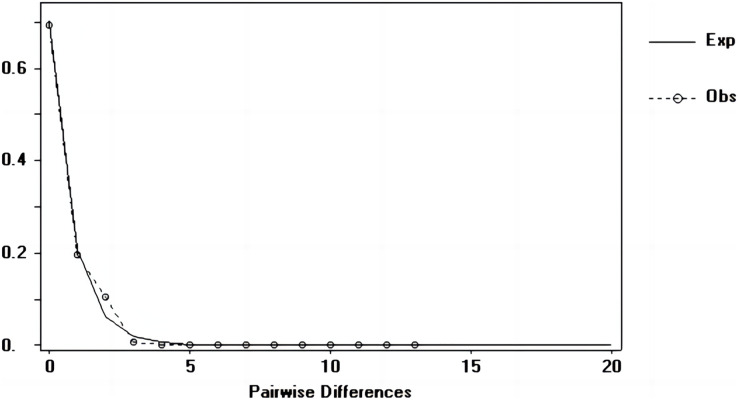
Nucleotide mismatch distributions of the COI marker used in this study.

Tajima’s D and Fu’s Fs values of each marker were shown in Table 7. Figures 6–8 showed the distribution diagram of nucleotide mismatches of three markers, all showing a single peak. Combining the results from Tajima’s D value, Fu’s Fs value and the nucleotide mismatch distribution map of each marker, we inferred that the breeding population of *C. mydas* in the Xisha (Paracel) Islands did not experience a population expansion.

**Table 7:** Neutral detection parameters of *Chelonia mydas* in Xisha (Paracel) Islands, asterisks (**) mean *p*-value < 0.01.

Marker	# Samples	Neutral detection	
		Tajima’s D	Fu’s Fs
D-loop	72	−2.60518**	−4.361
Cyt b	69	−2.35107**	−2.338
COI	71	−1.56483	−4.896

## Discussion

Gaillard et al. (2021) found that the breeding population of *C. mydas* from Xisha (Paracel) Islands represent a new geographic population with the unique D-loop haplotype CmP19, which is only sporadically found in other breeding or feeding grounds in the Indo-Pacific region. When combining the haplotypes identified by Gaillard et al. (2021) and Song et al. (2022), we found that the CmP19 haplotype represented 73% (*n* = 101) of the *C. mydas* breeding population in the Xisha (Paracel) Islands. Therefore, our study further confirms the uniqueness of this population. We discovered five new happlotypes and three haplotypes previously found in other areas (Dutton et al., 2014; Jensen et al., 2016; Joseph et al., 2016), increasing the number of D-loop haplotypes from the original seven to 15 in the Xisha (Paracel) population, which was much higher than the average (5.35, Table 4) of management units in Indo-Pacific region. Additionally, according to the D-loop haplotype network (Fig. 3), there are at least seven intermediary haplotypes may exist in nature, but these have not yet been sampled in this area. In this study, nine of the 15 D-loop haplotypes, three of the five Cyt b haplotypes, and two of the seven COI haplotypes represent only a single *C. mydas* sample. If more samples can be collected, a more reliable estimate of haplotype frequency can be provided. Thus, it is necessary to increase the sampling range and sample numbers in the future to find more potential haplotypes of the sea turtle population in this area.

In this study, the D-loop genetic diversity (haplotype diversity: 0.415, nucleotide diversity: 0.002) of *C. mydas* from the Xisha (Paracel) Islands is much smaller than that of Gaillard et al. (2021) and Song et al. (2022) (0.575, 0.009 and 0.628, 0.018, respectively). This could be due to the small sample sizes (16 and 13, respectively) in the previous two studies. Small sample sizes can greatly reduce resolution, the ability to discriminate between populations and the power to detect rare or unique haplotypes, which may lead to discrepancies between actual and observed gene richness (Marshall & Brown, 1975). Meanwhile, as the mean nesting interval for *C. mydas* is 10–17 days (Miller, 1997; Alvarado-Díaz, Arias-Coyotl & Delgado-Trejo, 2003), the possibility remains that hatchlings or dead embryos collected from different nests belong to the same female, thus making genetic diversity lower than it actually is in Xisha (Paracel) Islands. Although the number of samples has been greatly increased in our study, it is still small compared with other regions, such as the Galapagos Islands (Dutton et al., 2014 ). This is mainly because it is quite difficult to reach the Xisha (Paracel) Islands, and *C. mydas* breeding population is relatively small in this area.

Based on D-Loop haplotypes, Jensen et al. (2019) identified five evolutionary clades (clade III, IV, V, VII, and VIII) of *C. mydas* in the Indo-Pacific region, with clade VIII predominant. At present, individuals from the breeding population of the Xisha (Paracel) Islands were found in three of these clades (III, IV, VIII), with haplotypes in VIII dominating (73%), consistent with Jensen et al. (2019). The *C. mydas* population of the Xisha (Paracel) Islands shared four haplotypes with *C. mydas* from the Philippines, Malaysia, Indonesia, and Australia (CmP20, CmP47, CmP49, and CmP57). However, these four haplotypes only account for 8.9% of the total samples studied from the Xisha (Paracel) Islands (*n* = 101) and were relatively rare in this region. MtDNA shows only female-mediated gene flow, and will not actually show the contribution from males, who may be from outside the South China Sea. Therefore, further sampling, including more individuals and more nuDNA markers as well as telemetry data, will be needed to determine whether *C. mydas* in the Xisha (Paracel) Islands are communicating frequently with *C. mydas* from adjacent areas.

A reduction in population size often leads to a loss of genetic diversity, which reduces reproduction and survival (*e.g*., inbreeding depression) and further reduces population sizes (Lacy, 1997; Frankham, 2005). Sea turtle populations in the South China Sea have dropped sharply due to the massive illegal trade and habitat loss (Lin et al., 2021). The exploitation and trade of turtle products was common in Southeast Asia, especially in Malaysia, Indonesia, and the Philippines, where sea turtle populations have suffered from chronic illegal hunting (Dijk & Shepherd, 2004; Stiles, 2008; Lam et al., 2011). Gaillard et al. (2021) showed that the *C. mydas* recently confiscated in Hainan likely come from the Coral Triangle, and populations in the Xisha (Paracel) Islands and the Sulu Sea are facing serious illegal hunting pressure.The degradation of nesting beaches has also gradually reduced the breeding space of *C. mydas* in the Xisha (Paracel) Islands. For example, sea turtles used to lay eggs on Yongxing Island and Zhaoshu Island (Wei, 2016; Jia et al., 2019), but, due to human activities, they no longer go ashore there. In addition, marine pollution is threatening the remaining nesting beaches and breeding populations of sea turtles in the Xisha (Paracel) Islands. Beach debris, microplastic, and trace element pollution are widespread in the nesting grounds of *C. mydas* in the Xisha (Paracel) Islands, and those pollutants have potential negative impacts on the reproductive activities and embryo development of sea turtles (Zhang et al., 2020, 2021a; Jian et al., 2021; Zhang et al., 2022). Evidence of global warming was also recorded in the Xisha (Paracel) Islands, as beach temperatures have increased by 1–2 °C from 2018 to 2021 (Zhang et al., 2022). It is well-known that the sex of sea turtles is determined by the hatching temperature. The increase in hatching temperature will increase female offspring, which has been confirmed by laparoscopic surgery performed on sea turtles from South China Sea (Yeh et al., 2021). This gender imbalance will threaten reproduction and decrease turtle populations in the future (Stewart & Dutton, 2014). The positive feedback loop between small population size and low genetic diversity, termed “the vortex effect”, may ultimately lead to extinction (Gilpin & Soulé, 1986; Hughes et al., 2008).

Green sea turtles are a migratory marine species with a wide distribution range and are highly loyal to their breeding and feeding grounds (Formia et al., 2006; Hamabata, Kamezaki & Hikida, 2014). Confiscated *C. mydas* in the South China Sea affect not only the breeding population of *C. mydas* in the Xisha (Paracel) Islands, but also other distant nesting grounds, such as the Philippines and Malaysia (Gaillard et al., 2021; Zhang et al., 2021b). Therefore, it is necessary to strengthen cross-border cooperation in monitoring and protecting sea turtle populations in the South China Sea and internationally. Furthermore, it is necessary to completely prohibit the commercial use of sea turtles as live or wildlife products and to monitor market trends and trade routes to jointly protect sea turtle populations. The Xisha (Paracel) Islands are also a key area for Chinese fishermen who operate in the South China Sea (Wang et al., 2019). To strengthen the protection of sea turtles, the Chinese Government issued the “Sea Turtle Conservation Action Plan (2019–2033)” (Chinese Ministry of Agriculture & Rural Affairs, 2018) and upgraded all five sea turtle species from level II to level I on the “List of Wildlife under Special State Protection” of China in 2021 (Lin et al., 2021). These policies make the prospect of sea turtle protection brighter in the South China Sea. We recommend that a sea turtle sanctuary be established in the Xisha (Paracel) Islands to protect nesting and foraging sites, monitor nesting populations, and mitigate the effects of fishing and costal development on this population of *C. mydas*.

When analyzing the samples of the breeding population of *C. mydas* in the Xisha (Paracel) Islands, this study only analyzed the mitochondrial genes of *C. mydas*, which is relatively simple. In the later stage, it is urgent to combine nuclear genes, microsatellites, and other technologies (such as single nucleotide polymorphisms analysis or restriction site-associated DNA sequencing) to comprehensively evaluate the genetic diversity of the breeding populations of *C. mydas* in the Xisha (Paracel) Islands. Concurrently, it is necessary to further explore the feeding grounds of *C. mydas*, strictly plan fishing areas, and strengthen scientific outreach to communities for the public to effectively protect sea turtle populations in the South China Sea.

## Supplemental Information

10.7717/peerj.15115/supp-1 Supplemental Information 1 Sampling Information of *Chelonia mydas* in the Xisha Islands (Paracel).

10.7717/peerj.15115/supp-2 Supplemental Information 2 Construct the D-loop haplotype alignment sequence of the ML tree.

10.7717/peerj.15115/supp-3 Supplemental Information 3 ML tree of 26 D-loop haplotypes (384 bp) from rookeries of *C. mydas* in the Indo-Pacific and Japan regions, including 15 haplotypes from the Xisha Islands.

## Additional Information and Declarations

### DNA Deposition

The following information was supplied regarding the deposition of DNA sequences: Sequences are available at the National Center for Biotechnology information: OP320709–OP320723, OK284742, OK324138.

### Data Availability

The following information was supplied regarding data availability: The data is available in the Supplemental Files.
