# Supplementary material for: Revisiting the genetic diversity and population structure of the endangered Green Sea Turtle (Chelonia mydas) breeding populations in the Xisha (Paracel) Islands, South China Sea
Source: PeerJ. 2023 Mar 22;11:e15115. doi: 10.7717/peerj.15115 (PMC10039654; doi:10.7717/peerj.15115)
Supplement: Supplemental Information 1 [file peerj-11-15115-s001.docx]

| **Sampling year** | **Sampling site** | | **Sample number** | **Sample source** |
| --- | --- | --- | --- | --- |
| 2017 | Xuande Island | North Island | 1 | Female |
|  |  | North Island | 2 | Female |
|  |  | North Island | 3 | Female |
|  |  | North Island | 4 | Female |
|  |  | North Island | 5 | Female |
|  | Yongle Island | Ganquan Island | 6 | Dead hatchling |
|  |  | Ganquan Island | 7 | Dead hatchling |
|  |  | Ganquan Island | 8 | Hatchling |
|  |  | Ganquan Island | 9 | Hatchling |
|  |  | Ganquan Island | 10 | Hatchling |
|  |  | Ganquan Island | 11 | Juvenile |
|  |  | Ganquan Island | 12 | Hatchling |
|  |  | Ganquan Island | 13 | Hatchling |
|  |  | Ganquan Island | 14 | Hatchling |
|  |  | Ganquan Island | 15 | Hatchling |
| 2018 | Xuande Island | North Island | 16 | Female |
|  |  | North Island | 17 | Female |
|  |  | North Island | 18 | Female |
|  |  | South Island | 19 | Hatchling |
|  |  | North Island | 20 | Hatchling |
|  |  | North Island | 21 | Hatchling |
|  |  | North Island | 22 | Hatchling |
|  |  | North Island | 23 | Hatchling |
|  |  | North Island | 24 | Hatchling |
|  |  | North Island | 25 | Hatchling |
|  |  | North Island | 26 | Hatchling |
|  |  | North Island | 27 | Hatchling |
|  |  | North Island | 28 | Hatchling |
|  |  | North Island | 29 | Hatchling |
|  |  | North Island | 30 | Hatchling |
|  |  | North Island | 31 | Hatchling |
|  |  | North Island | 32 | Hatchling |
|  |  | North Island | 33 | Hatchling |
|  |  | North Island | 34 | Hatchling |
|  |  | North Island | 35 | Juvenile |
|  |  | North Island | 36 | Hatchling |
|  |  | North Island | 37 | Hatchling |
|  |  | North Island | 38 | Hatchling |
|  |  | North Island | 39 | Hatchling |
|  |  | North Island | 40 | Hatchling |
|  |  | North Island | 41 | Hatchling |
| 2020 | Xuande Island | North Island | 42 | Female |
|  |  | North Island | 43 | Female |
|  |  | North Island | 44 | Female |
|  |  | North Island | 45 | Female |
|  |  | North Island | 46 | Female |
|  |  | North Island | 47 | Hatchling |
|  |  | North Island | 48 | Hatchling |
|  |  | North Island | 49 | Hatchling |
|  |  | North Island | 50 | Hatchling |
|  |  | North Island | 51 | Hatchling |
|  |  | South Island | 52 | Hatchling |
|  |  | North Island | 53 | Hatchling |
|  |  | South Island | 54 | Hatchling |
|  |  | North Island | 55 | Hatchling |
| 2021 | Xuande Island | North Island | 56 | Female |
|  |  | North Island | 57 | Female |
|  |  | North Island | 58 | Female |
|  |  | North Island | 59 | Female |
|  |  | North Island | 60 | Female |
|  |  | North Island | 61 | Female |
|  |  | North Island | 62 | Female |
|  |  | North Island | 63 | Female |
|  |  | North Island | 64 | Female |
|  |  | South Sand | 65 | Dead embryo |
|  |  | West Sand | 66 | Dead embryo |
|  |  | North Island | 67 | Dead embryo |
|  |  | North Island | 68 | Dead embryo |
|  |  | North Island | 69 | Dead embryo |
|  |  | North Island | 70 | Dead embryo |
|  | Yongle Island | Jinqing Island | 71 | Dead embryo |
|  |  | Jinqing Island | 72 | Dead embryo |
