# Supplementary figures and images for: Revisiting the genetic diversity and population structure of the endangered Green Sea Turtle (Chelonia mydas) breeding populations in the Xisha (Paracel) Islands, South China Sea
Source: PeerJ. 2023 Mar 22;11:e15115. doi: 10.7717/peerj.15115 (PMC10039654; doi:10.7717/peerj.15115)

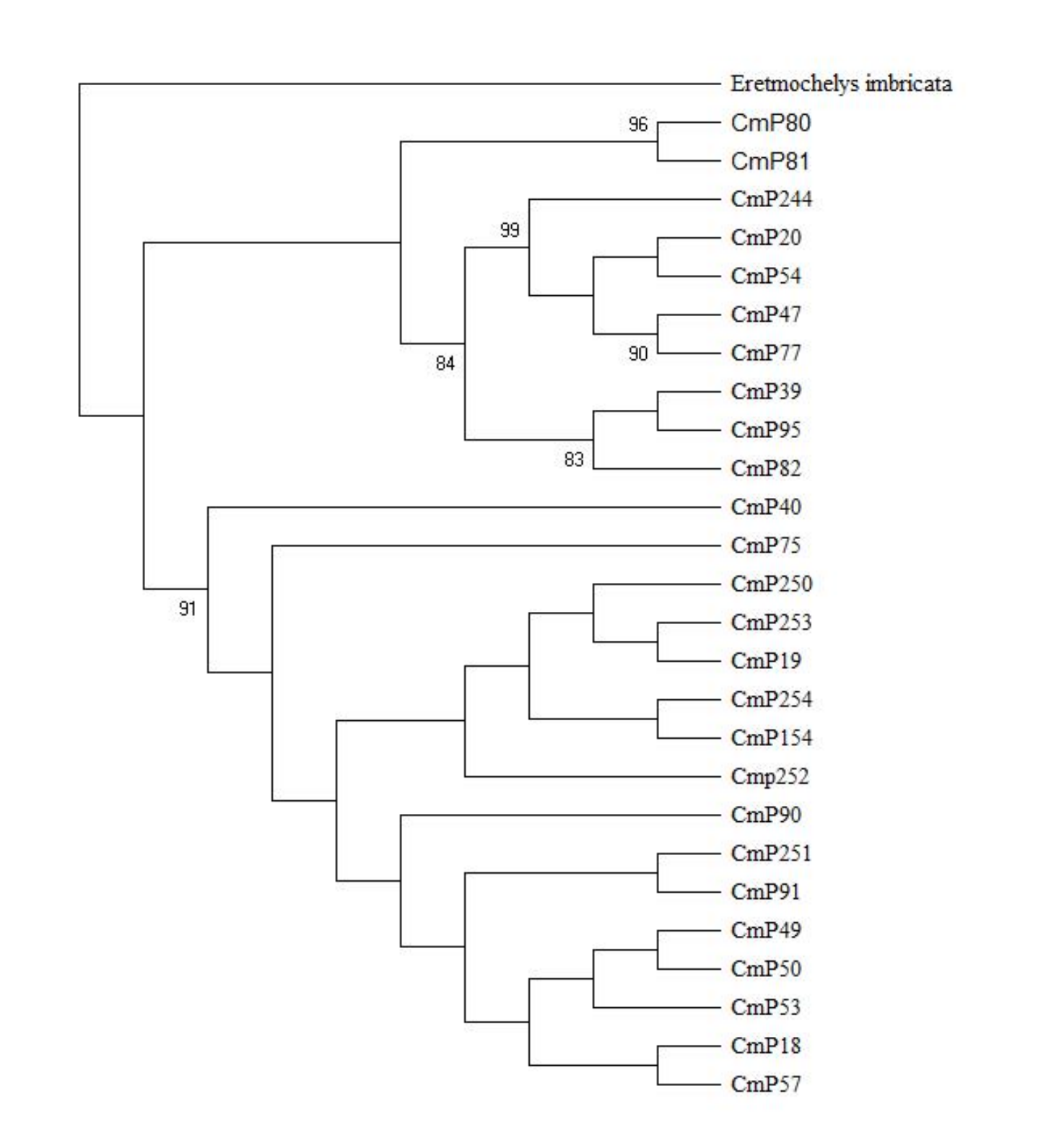

Supplement: Supplemental Information 3 [file peerj-11-15115-s003.png]
